# The Implications of Endoscopic Ulcer in Early Gastric Cancer: Can We Predict Clinical Behaviors from Endoscopy?

**DOI:** 10.1371/journal.pone.0164339

**Published:** 2016-10-14

**Authors:** Yoo Jin Lee, Jie-Hyun Kim, Jae Jun Park, Young Hoon Youn, Hyojin Park, Jong Won Kim, Seung Ho Choi, Sung Hoon Noh

**Affiliations:** 1 Department of Internal Medicine, Gangnam Severance Hospital, Yonsei University College of Medicine, Seoul, Korea; 2 Department of Surgery, Gangnam Severance Hospital, Yonsei University College of Medicine, Seoul, Korea; 3 Department of Surgery, Yonsei University College of Medicine, Seoul, Korea; University Hospital Llandough, UNITED KINGDOM

## Abstract

**Background:**

The presence of ulcer in early gastric cancer (EGC) is important for the feasibility of endoscopic resection, only a few studies have examined the clinicopathological implications of endoscopic ulcer in EGC.

**Objectives:**

To determine the role of endoscopic ulcer as a predictor of clinical behaviors in EGC.

**Methods:**

Data of 3,270 patients with EGC who underwent surgery between January 2005 and December 2012 were reviewed. Clinicopathological characteristics were analyzed in relation to the presence and stage of ulcer in EGC. Based on endoscopic findings, the stage of ulcer was categorized as active, healing, or scar. Logistic regression analysis was performed to analyze factors associated with lymph node metastasis (LNM).

**Results:**

2,343 (71.7%) patients had endoscopic findings of ulceration in EGC. Submucosal (SM) invasion, LNM, lymphovascular invasion (LVI), perineural invasion, and undifferentiated-type histology were significantly higher in ulcerative than non-ulcerative EGC. Comparison across different stages of ulcer revealed that SM invasion, LNM, and LVI were significantly associated with the active stage, and that these features exhibited significant stage-based differences, being most common at the active stage, and least common at the scar stage. The presence of endoscopic ulcer and active status of the ulcer were identified as independent risk factors for LNM.

**Conclusions:**

Ulcerative EGC detected by endoscopy exhibited more aggressive behaviors than non-ulcerative EGC. Additionally, the endoscopic stage of ulcer may predict the clinicopathological behaviors of EGC. Therefore, the appearance of ulcers should be carefully evaluated to determine an adequate treatment strategy for EGC.

## Introduction

Endoscopic resection (ER) has become a standard treatment for patients with early gastric cancer (EGC) without risk of lymph node metastasis (LNM). It has the advantages of being less invasive and more effective than surgery [1, 2]. To determine a proper management strategy, clinicians should be able to predict the clinical behaviors of EGC through accurate preoperative endoscopic examination. Currently, ER for EGC is generally chosen based on the Japanese gastric cancer treatment guidelines [3]. The traditionally accepted indication for ER has been limited to differentiated intramucosal cancer less than 20 mm in diameter without ulceration [4]. However, studies have suggested that select patients with EGC can also be considered candidates for ER with a low risk of LNM [4, 5]. Thus, the indications for ER have been expanded as follows: 1) differentiated intramucosal cancer > 20 mm in size without ulceration; 2) differentiated intramucosal cancer ≤ 30 mm with ulceration; and 3) undifferentiated intramucosal cancer ≤ 20 mm without ulceration [3].

Although the presence of ulcer has been included in the expanded criteria based on the low risk of LNM, the clinicopathological implications of ulcer on EGC have not been recently evaluated [4]. Moreover, it is notable that the ER criteria refer to histologic ulcer rather than endoscopic ulcer, even though in actual clinical practice, the treatment strategy is determined based on preoperative evaluation including endoscopic appearance. However, the clinical significance of endoscopic ulcer in EGC remains unclear. In addition, no study to date has investigated the clinical behavior of EGC depending on the stage of ulcer. Therefore, this study aimed to validate the role of endoscopic ulcer on the clinical behavior of EGC. We also evaluated whether the stage of ulcer is related to the clinicopathological behavior of EGC. A greater understanding of the clinical implications of ulcerative EGC would be helpful in enhancing decision making with regard to treatment strategy.

## Materials and Methods

The Institutional Review Board (IRB) of Severance and Gangnam Severance Hospital approved this study (4-2012-0472). This study was retrospective, we received a consent exemption from the IRB.

### Patients

A total of 3,357 patients with EGC underwent gastrectomy at Severance and Gangnam Severance hospital between January 2005 and December 2012. We excluded 87 patients whose endoscopic photos were too poor to characterize the lesion or for whom clinicopathological data were not available. Finally, data from 3,270 patients were retrospectively reviewed. Patients’ demographic characteristics, such as age and gender, were obtained from medical records.

### Endoscopic evaluation

Endoscopic images including the presence of ulcer and ulcer stage were independently reviewed by two experienced endoscopists blinded to the clinicopathological data. When the interpretations showed a discrepancy, the final diagnosis was determined by consensus between the two endoscopists after discussion.

**Fig 1:**
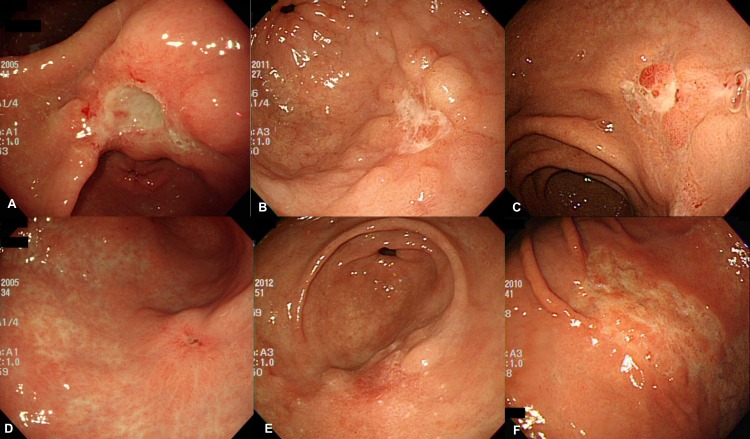
Endoscopic appearance of stages of ulcer in EGC. **(A)** A1 stage **(B)** A2 stage **(C)** H1 stage **(D)** H2 stage **(E)** S1 stage **(F)** S2 stage.

Ulcerative EGC was defined when EGC presented with endoscopic ulcer. Based on previous reports, endoscopic ulcer was defined as follows: i) depressed lesion covered with an exudative base of more than 1 cm, ii) sharply demarcated and raised margin, and iii) surrounding mucosal edema or fold convergence. Ulcer scar was defined as a slightly depressed or flat lesion coexisting with reddish regenerating epithelium of more than 1 cm and a surrounding edematous mucosa or convergence of fold [6]. The stages of ulcer in EGC were categorized as active (A), healing (H), or scar (S) stage, using a previously proposed six-stage system [7]. The representative endoscopic appearance of stages of ulcer in EGC is depicted in Fig 1.

### Histologic evaluation

By reviewing the pathology report from the gastrectomy specimen, we assessed the following: the location and gross type of the tumor, depth of invasion, lymphovascular invasion (LVI), perineural invasion (PNI), and LNM. Histological classifications including the Lauren and Japanese classifications were also reviewed. Tumor size was determined pathologically, based on the longest diameter of the tumor. Histology was based on the criteria of the Japanese classification of gastric carcinoma [8]. The tumor location was classified with respect to the longitudinal axis of the stomach as upper, middle, or lower third. The gross types of tumor were classified as elevated, flat, and depressed types. Depth of tumor invasion was grouped into intramucosal and submucosal lesions.

### Statistical analysis

The data are presented as case numbers (%). The associations between diverse categorical variables were evaluated using Chi-squared tests or Fisher’s exact tests. To identify factors associated with LNM, logistic regression analysis was performed. All factors that reached significance in the univariate analysis were included in the multivariate analysis. A two-tailed *P* value of <0.05 was considered statistically significant. Statistical analysis was performed using SPSS software (Ver. 20.0; IBM Corp., Armonk, New York, USA).

## Results

### Baseline characteristics

Of the 3,270 patients, ulcer was found in 2,343 (71.7%). Among patients with ulcerative EGC, the healing stage of ulcer was the most frequent, followed by the active stage. Undifferentiated-type histology was identified in 1,601 patients (49.0%). About 48% of patients had submucosally invasive cancer. The incidence of LNM was 10.5% (Table 1).

**Table 1:** The Clinicopathologic Characteristics of the Subjects (n = 3,270).

Characteristics	No. of patients (n, %)
**Male**	2,117 (64.7)
**Age (year)**	
≤40	294 (9.0)
>40	2,976 (91.0)
**Tumor location**	
Upper	345 (10.6)
Middle	602 (18.4)
Lower	2,323 (71.0)
**Ulcer**	
Absence	927 (28.3)
Presence	2,343 (71.7)
**Ulcer stage**	
A1	174 (7.4)
A2	519 (22.2)
H1	656 (28.0)
H2	725 (30.9)
S1	209 (8.9)
S2	60 (2.6)
**Gross type**	
Elevated	615 (18.8)
Flat	926 (28.3)
Depressed	1,729 (52.9)
**Lauren classification**	
Intestinal	1,839 (56.2)
Diffuse	1,263 (38.6)
Mixed	168 (5.1)
**Japanese classification**	
Differentiated	1,669 (51.0)
Undifferentiated	1,601 (49.0)
**Tumor diameter (mm)**	
<30	2,203 (67.4)
≥20	1,067 (32.6)
**Depth of invasion**	
Mucosa	1,715 (52.4)
Submucosa	1,555 (47.6)
**Lymphovascular invasion**	388 (11.9)
**Perineural invasion**	77 (2.4)
**Lymph node metastasis**	344 (10.5)

### Clinicopathological characteristics in relation to the presence of endoscopic ulcer in EGC

Table 2 compares the clinicopathological features of EGC in ulcerative and non-ulcerative EGC. Diffuse-type histology in the Lauren classification and undifferentiated-type histology in the Japanese classification were significantly associated with ulcerative EGC compared to non-ulcerative EGC. Ulcerative EGC was also significantly associated with SM invasion, LVI, PNI, and LNM, which are known to be poor prognostic factors in EGC. A greater number of ulcerative EGC cases relative to non-ulcerative EGC cases presented in the lower third of the stomach and were of the depressed gross type.

**Table 2:** Clinicopathologic Characteristics in Relation to the Presence of Endoscopic Ulcer in Early Gastric Cancer.

Characteristics	Ulcer (n, %)		*P*
	Presence	Absence	
**Male**	1,530 (65.3)	587 (63.3)	0.286
**Age (year) >40**	2,118 (90.4)	858 (92.6)	0.052
**Tumor location**			**0.014**
Upper	224 (9.6)	121 (13.1)	
Middle	435 (18.6)	167 (18.0)	
Lower	1,684 (71.9)	639 (68.9)	
**Gross type**			**< 0.001**
Elevated	238 (10.2)	377 (40.7)	
Flat	430 (18.4)	496 (53.5)	
Depressed	1,675 (71.5)	54 (5.8)	
**Lauren classification**			**0.001**
Intestinal	1,277 (54.5)	562 (60.6)	
Diffuse	930 (39.7)	333 (35.9)	
Mixed	136 (5.8)	32 (3.5)	
**Japanese classification**			**< 0.001**
Differentiated	1,143 (48.8)	526 (56.7)	
Undifferentiated	1,200 (51.2)	401 (43.3)	
**Tumor diameter (mm) ≥ 30**	759 (32.4)	308 (33.2)	0.648
**Depth of invasion**			**< 0.001**
Mucosa (T1a)	1,129 (48.2)	586 (63.2)	
Submucosa (T1b)	1,214 (51.8)	341 (36.8)	
**Lymphovascular invasion**	327 (14.0)	61 (6.6)	**< 0.001**
**Perineural invasion**	76 (3.2)	1 (0.1)	**< 0.001**
**Lymph node metastasis**	294 (12.5)	50 (5.4)	**<0.001**

Biological behavior was analyzed in relation to the presence of ulcer in each subgroup of differentiated and undifferentiated-type EGC. Ulcerative EGC exhibited higher rates of SM invasion, LVI, PNI, and LNM than non-ulcerative EGC in both subgroups (S1 and S2 Tables).

### Clinicopathological characteristics in relation to endoscopic stage of ulcer in EGC

Among the 2,343 patients with ulcerative EGC, the proportions of active, healing, and scar stages were 29.6%, 58.9%, and 11.5%, respectively. Table 3 presents clinicopathological factors in relation to endoscopic stage of ulcer. Interestingly, EGC larger than 30 mm, SM invasion, LVI, and LNM were significantly associated with ulcer stage, being most common in the active stage and least common in the scar stage. However, diffuse- and undifferentiated-type histology showed an inverse correlation with ulcer stage, being most common in the scar stage and least common in the active stage. We performed subgroup analysis of endoscopic ulcer stage in relation to histological differentiation based on the Japanese classification. In differentiated-type EGC, SM invasion and LNM were significantly associated with ulcer stage, being most common in the active stage and least common in the scar stage (S3 Table). Similarly, these poor prognostic factors were also significantly associated with ulcer stage in undifferentiated-type EGC, exhibiting the same general pattern (S4 Table).

**Table 3:** Clinicopathologic Factors in Relation to Endoscopic Stage of Ulcer in Early Gastric Cancer (n = 2,343).

Characteristics	Ulcer stage			*P*
	Active (n = 693)	Healing (n = 1,381)	Scar (n = 269)	
**Male, n (%)**	486 (70.1)	881 (63.8)	162 (60.2)	**0.003**
**Age >40 years, n (%)**	627 (90.5)	1,242 (89.9)	248 (92.2)	0.513
**Tumor location, n (%)**				**0.024**
Upper	67 (9.7)	121 (8.8)	36 (13.4)	
Middle	124 (17.9)	249 (18.0)	62 (23.0)	
Lower	502 (72.4)	1,011 (73.2)	171 (63.6)	
**Gross type, n (%)**				**0.001**
Elevated	78 (11.3)	145 (10.5)	14 (5.2)	
Flat	109 (15.7)	253 (18.3)	69 (25.7)	
Depressed	506 (73.0)	983 (71.2)	186 (69.1)	
**Lauren classification, n (%)**				**0.003**
Intestinal	407 (58.7)	746 (54.0)	123 (45.7)	
Diffuse	244 (35.2)	562 (40.7)	125 (46.5)	
Mixed	42 (6.1)	73 (5.3)	21 (7.8)	
**Japanese classification, n (%)**				**0.001**
Differentiated	366 (52.8)	669 (48.4)	107 (39.8)	
Undifferentiated	327 (47.2)	712 (51.6)	162 (60.2)	
**Tumor diameter ≥30 mm, n (%)**	246 (35.5)	447(32.4)	67 (24.9)	**0.007**
**Depth of invasion, n (%)**				**< 0.001**
Mucosa (T1a)	231 (33.3)	711 (51.5)	187 (69.5)	
Submucosa (T1b)	462 (66.7)	670 (48.5)	82 (30.5)	
**Lymphovascular invasion, n (%)**	118 (17.0)	182 (13.2)	26 (9.7)	**0.006**
**Perineural invasion, n (%)**	25 (3.6)	46 (3.3)	5 (1.9)	0.373
**Lymph node metastasis, n (%)**	125 (18.0)	155 (11.2)	14 (5.2)	**< 0.001**

### Clinicopathological factors associated with lymph node metastasis

LNM was observed in 344 (10.5%) of the 3,270 patients. Logistic regression analysis revealed that the presence of ulcer, active stage of ulcer, healing stage of ulcer, tumor size ≥30 mm, SM invasion, and LVI were independent risk factors for LNM in the EGC patients overall (n = 3,270) (Tables 4 and 5). When we categorized the patients into two subgroups—those with differentiated-type and undifferentiated-type EGC—the proportion of LNM was 10.7% in differentiated-type and 10.4% in undifferentiated-type EGC. In differentiated-type EGC, multivariate analysis revealed that elevated gross type, active stage of ulcer, SM invasion, and LVI were independent risk factors for LNM (S5 Table). In undifferentiated-type EGC, the presence of ulcer, active stage of ulcer, tumor size ≥30 mm, SM invasion, and LVI were identified as independent risk factors for LNM (S6 Table).

**Table 4:** Univariate Analysis of Risk Factors for Lymph Node Metastasis (n = 3,270).

N (%)	Lymph node metastasis		*P*	N (%) (cont’d)	Lymph node metastasis		*P*
	Presence n = 344	Absence n = 2,926			Presence n = 344	Absence n = 2,926	
**Age > 40 (year)**	304 (88.4)	2,672 (91.3)	0.071	**Gross type**			**< 0.001**
**Male**	220 (64.0)	1.897 (64.8)	0.747	Elevated	88 (25.6)	527 (18.0)	
**Tumor location**			0.750	Flat	56 (16.3)	870 (29.7)	
Upper	33 (9.6)	312 (10.7)		Depressed	200 (58.1)	1,529 (52.3)	
Middle	61 (17.7)	541 (18.5)		**Lauren classification**			**<0.001**
Lower	250 (72.7)	2,073 (70.8)		Intestinal	203 (59.0)	1,636 (55.9)	
**Ulcer**			**< 0.001**	Diffuse	110 (32.0)	1,153 (39.4)	
Presence	294 (85.5)	2,049 (70.0)		Mixed	31 (9.0)	137 (4.7)	
Absence	50 (14.5)	877 (30.0)		**Tumor diameter ≥ 30 (mm)**	165 (48.0)	902 (30.8)	**< 0.001**
**Ulcer stage**			**< 0.001**	**Depth of invasion**			**<0.001**
Active stage	125 (42.5)	568 (27.7)		Mucosa	40 (11.6)	1,675 (57.2)	
Healing stage	155 (52.7)	1,226 (59.8)		Submucosa	304 (88.4)	1,251 (42.8)	
Scar stage	14 (4.8)	255 (12.4)		**Lymphovascular invasion**	177 (51.5)	211 (7.2)	**<0.001**
				**Perineural invasion**	18 (5.2)	59 (2.0)	**<0.001**

**Table 5:** Multivariate Analysis of Risk Factors for Lymph Node Metastasis (n = 3,270).

	Logistic regression model (including ulcer)			Logistic regression model (including ulcer stage)		
N (%)	Odds ratio	95% CI	*P*	Odds ratio	95% CI	*P*
**Age >40 (year)**						
**Male**						
**Tumor location**						
Upper						
Middle						
Lower						
**Ulcer**				-		
Presence	1.730	1.169–2.559	**0.006**			
Absence	1					
**Ulcer stage**	-					
Active stage	-			2.664	1.423–4.988	**0.002**
Healing stage	-			1.858	1.006–3.430	**0.048**
Scar stage	-			1		
**Gross type**						
Elevated	1.477	0.983–2.221	0.061	1.352	0.828–2.205	0.228
Flat	1			1		
Depressed	1.347	0.944–1.923	0.101	1.134	0.775–1.658	0.517
**Lauren classification**						
Intestinal	0.863	0.652–1.143	0.304	0.792	0.587–1.069	0.128
Diffuse	1			1		
Mixed	1.364	0.819–2.274	0.233	1.353	0.793–2.310	0.267
**Tumor diameter ≥ 30 (mm)**	1.566	1.212–2.022	**0.001**	1.636	1.240–2.158	**< 0.001**
**Depth of invasion**						
Mucosa	1			1		
Submucosa	5.077	3.529–7.302	**<0.001**	4.262	2.873–6.323	**< 0.001**
**Lymphovascular invasion**	6.900	5.245–9.078	**< 0.001**	6.095	4.511–8.234	**< 0.001**
**Perineural invasion**	0.946	0.520–1.722	0.856	0.910	0.494–1.676	0.761

## Discussion

With advances in endoscopic techniques, ER for EGC has increasingly been used. Studies have demonstrated that ER is comparable to surgery with respect to long-term outcomes, and that it confers superior quality of life compared to surgery [5, 9]. To select appropriate candidates for ER, a precise pretreatment prediction of the biological behavior of EGC is necessary. Thus, numerous studies have set out to predict clinical characteristics of EGC by analyzing various clinicopathological factors (e.g., depth of invasion, tumor size, endoscopic gross appearance, and histological differentiation) [10, 11] and imaging modalities (e.g., endoscopic ultrasonography and narrow band imaging with magnifying endoscopy) [12, 13].

Since Gotoda et al. reported that LNM was significantly higher in ulcerative EGC (3.4%) than in non-ulcerative EGC (0.5%), the presence of ulcer has been recognized as crucial for determining an optimal treatment strategy for EGC [4]. In principle, the presence of ulcer in current ER criteria does not refer to endoscopic ulcer but to histologic ulcer, because the criteria were based on data from surgically resected specimens [4]. Also, recent data identified that histologic ulcer was related to LNM of EGC, emphasizing the importance of histologic ulcer for predicting LNM of EGC [14]. However, this poses a problem because there is no proper method for histologically identifying the presence of ulcer prior to ER. Although histologic ulcer could be helpful to select patients who need additional surgery after ER, endoscopic ulcer is more crucial to predict the risk of LNM than histologic ulcer when determining an optimal management strategy in clinical practice. Thus, to date, clinicians in community practice have relied on endoscopic findings of ulcer in the decision-making process. Most published studies also adopt the endoscopic presence of ulcer to evaluate the outcomes and feasibility of ER for EGC [15–17]. However, pathologists and endoscopists may differ substantially with regard to the presence of ulcer; a recent study reported that the presence of histologic ulcer was unclear in some surgically resected EGC samples despite the definite presence of an ulcer as determined endoscopically [6]. It can be argued that the definition of histological ulcer is more objective and reliable than that of endoscopic one. Malignant cycle of ulcer which means the improvement and exacerbation of ulceration in EGC [18], the ambiguous definition of endoscopic ulcer, and inter- and intraobserver variation among endoscopists could be potential limitations of endoscopic ulcer. However, the treatment of EGC is usually performed without delay regardless of malignant cycle in clinical practice.

Hence, it is important to identify the clinical significance of ulcer in EGC on the basis of endoscopic findings. However, due to a lack of data, the implications of endoscopic ulcer and ulcer stages on the biological behavior of EGC are largely unknown. Therefore, the current study aimed to evaluate the role of endoscopic ulcer as a prognosticator of EGC.

This study revealed that SM invasion, LNM, LVI, PNI, and undifferentiated-type histology including poorly differentiated adenocarcinoma and signet ring cell carcinoma were significantly higher in ulcerative EGC than in non-ulcerative EGC. Because SM invasion, LNM, LVI, PNI, and undifferentiated-type histology are important prognostic parameters that reflect the biological behaviors of EGC, prediction of these parameters provides useful information for decision-making with regard to the treatment of ulcerative EGC. The underlying mechanisms for the aggressive behaviors of ulcerative EGC presented in this study require further investigation.

One of the valuable findings of the present study is that the stage of ulcer was significantly associated with SM invasion, LNM, and LVI. These features were most frequently observed in the active stage, followed by the healing stage, and finally, the scar stage of ulcer in EGC. Our findings indicate that the stage of ulcer also reflects the clinicopathological behaviors of EGC. The ulcer stage in EGC is usually overlooked during the endoscopic examination because it is not included in the ER criteria. However, as clinicians, we sometimes raise practical questions when we encounter the healing or scar stage of ulcer in EGC, inquiring whether the presence of ulcer at these stages is as pertinent as ulcer at the active stage. To the best of our knowledge, this is the first report to demonstrate the impact of ulcer stage on clinicopathological behaviors in EGC. Based on these results, we suggest that endoscopists consider documenting ulcer stage in endoscopic reports.

It is notable that the presence of endoscopic ulcer and active ulcer stage were independent risk factors for LNM in logistic regression analysis. Because low risk of LNM is the most important precondition for ER in treating EGC, these data may be useful when ulcerative EGC is encountered in routine clinical practice. In addition, the elevated gross type of EGC was an independent risk factor for LNM in differentiated-type gastric cancer. Considering the clinical significance of endoscopic gross appearance and ulceration on clinicopathological characteristics of EGC, more precise endoscopic examination is necessary during diagnostic endoscopy with conventional white-light endoscopy. Furthermore, endoscopists should strive for appropriate descriptions of their findings in endoscopy reports with regard to both gross appearance and ulceration.

This study has certain limitations. First, we did not evaluate the cycle of a malignant ulcer that has been identified in 29% of patients with EGC.[19]. Similarly, we did not assess whether patients had been treated with antisecretory medication. Previous studies have reported that some patients prescribed antisecretory agents showed improvement of malignant gastric ulcers [19, 20]. However, the decision to perform ER for EGC is usually at the discretion of individual endoscopists at the time of endoscopy. It is not routine in clinical practice to delay decisions regarding treatment of EGC to take into account the malignant cycle. Thus, we suggest that our findings provide valuable implications regardless of the malignant ulcer cycle in EGC in real-life situations. Second, the endoscopic definition of ulcer is not yet clearly established, especially in relation to the specification of the lower limit of ulcer size, but its pathological definition is straightforward [17, 21]. This potentially limits the accuracy of the assessment of endoscopic ulcers, which presents a common dilemma for investigators [19, 21]. Although, the endoscopic images were reviewed and corrected by two experienced endoscopists based on the prior reports, we did not assessed interobserver agreement with stage determination. Thus, further study is necessary to evaluate inter-observer agreement with regard to the endoscopic classification of ulcer. Third, the proportion of ulcerative EGC cases in this study is slightly higher than that reported in other studies [4, 22]. This disparity may be due to differences in the enrolment of patients. We examined patients who underwent surgery, not ER. It may be possible that surgery tends to be recommended for patients with ulcerative EGC more frequently than for patients with non-ulcerative EGC, despite the expanded criteria. Additionally, there may have been selective reporting limited to active ulcers in previous studies indicating a very low rate of ulcer. Fourth, the patients in this retrospective study may not be a representative sample of EGC patients, since our evaluation was limited to patients who underwent gastrectomy in two hospitals in South Korea. The results await further confirmation through multicenter prospective trials with larger sample sizes.

In conclusion, ulcerative EGC displayed more aggressive behaviors than non-ulcerative EGC with respect to SM invasion, LNM, LVI, and, PNI. When compared in relation to stages of ulcer, these poor prognostic factors were significantly associated with the active ulcer stage, indicating that the stage of ulcer may predict the clinicopathological behaviors of EGC. Meticulous examination of endoscopic appearance of ulcers in EGC is essential before treatment decisions are made.

## Supporting Information

S1 Table Biologic behaviors according to presence of ulcer in differentiated-type early gastric cancer (n = 1,669). (DOCX)

S2 Table Biologic behaviors according to presence of ulcer in undifferentiated-type early gastric cancer (n = 1,601). (DOCX)

S3 Table Biologic behaviors according to the stage of ulcer in differentiated-type early gastric cancer (n = 1,142). (DOCX)

S4 Table Biologic behaviors according to the stage of ulcer in undifferentiated-type early gastric cancer (n = 1,201). (DOCX)

S5 Table Univariate and multivariate analysis of risk factors for lymph node metastasis in differentiated-type gastric cancer (n = 1,669). (DOCX)

S6 Table Univariate and multivariate analysis of risk factors for lymph node metastasis in undifferentiated-type gastric cancer (n = 1,601). (DOCX)
