# Supplementary material for: The Implications of Endoscopic Ulcer in Early Gastric Cancer: Can We Predict Clinical Behaviors from Endoscopy?
Source: PLoS One. 2016 Oct 14;11(10):e0164339. doi: 10.1371/journal.pone.0164339 (PMC5065238; doi:10.1371/journal.pone.0164339)
Supplement: S1 Table — (DOCX) [file pone.0164339.s001.docx]

**S1 table.** Biologic behaviors according to presence of ulcer in differentiated-type early gastric cancer (n = 1,669)

|  | Ulcer (n, %) | | *P* |
| --- | --- | --- | --- |
|  | Presence | Absence |  |
| Depth of invasion |  |  | **< 0.001** |
| Mucosa (T1a) | 484 (42.3) | 302 (57.4) |  |
| Submucosa (T1b) | 659 (57.7) | 224 (42.6) |  |
| Lymphovascular invasion | 176 (15.4) | 49 (9.3) | **0.001** |
| Perineural invasion | 27 (2.4) | 1 (0.2) | **0.001** |
| Lymph node metastasis | 143(12.5) | 35 (6.7) | **< 0.001** |
