# Supplementary material for: The Implications of Endoscopic Ulcer in Early Gastric Cancer: Can We Predict Clinical Behaviors from Endoscopy?
Source: PLoS One. 2016 Oct 14;11(10):e0164339. doi: 10.1371/journal.pone.0164339 (PMC5065238; doi:10.1371/journal.pone.0164339)
Supplement: S2 Table — (DOCX) [file pone.0164339.s002.docx]

**S2 table.** Biologic behaviors according to presence of ulcer in undifferentiated-type early gastric cancer (n = 1,601)

|  | Ulcer (n, %) | | *P* |
| --- | --- | --- | --- |
|  | Presence | Absence |  |
| Depth of invasion |  |  | **<0.001** |
| Mucosa (T1a) | 645 (53.8) | 284 (70.8) |  |
| Submucosa (T1b) | 555 (46.2) | 117 (29.2) |  |
| Lymphovascular invasion | 151 (12.6) | 12 (3.0) | **<0.001** |
| Perineural invasion | 49 (4.1) | 0 | **< 0.001** |
| Lymph node metastasis | 151 (12.6) | 15 (3.7) | **< 0.001** |
