# Supplementary material for: The Implications of Endoscopic Ulcer in Early Gastric Cancer: Can We Predict Clinical Behaviors from Endoscopy?
Source: PLoS One. 2016 Oct 14;11(10):e0164339. doi: 10.1371/journal.pone.0164339 (PMC5065238; doi:10.1371/journal.pone.0164339)
Supplement: S3 Table — (DOCX) [file pone.0164339.s003.docx]

**S3 table.** Biologic behaviors according to the stage of ulcer in differentiated-type early gastric cancer (n = 1,142)

|  | Ulcer stage (n, %) | | | *P* value |
| --- | --- | --- | --- | --- |
|  | Active | Healing | Scar |  |
| Depth of invasion |  |  |  | **<0.001** |
| Mucosa (T1a) | 113 (30.9) | 303 (45.3) | 67 (62.6) |  |
| Submucosa (T1b) | 253 (69.1) | 366 (54.7) | 40 (37.4) |  |
| Lymphovascular invasion | 68 (18.6) | 96 (14.3) | 11 (10.3) | 0.062 |
| Perineural invasion | 10 (2.7) | 17 (2.5) | 0 | 0.235 |
| Lymph node metastasis | 67 (18.3) | 70 (10.5) | 5 (4.7) | **<0.001** |
