# Supplementary material for: The Implications of Endoscopic Ulcer in Early Gastric Cancer: Can We Predict Clinical Behaviors from Endoscopy?
Source: PLoS One. 2016 Oct 14;11(10):e0164339. doi: 10.1371/journal.pone.0164339 (PMC5065238; doi:10.1371/journal.pone.0164339)
Supplement: S4 Table — (DOCX) [file pone.0164339.s004.docx]

**S4 table.** Biologic behaviors according to the stage of ulcer in undifferentiated-type early gastric cancer (n = 1,201)

|  | Ulcer stage (n, %) | | | *P* value |
| --- | --- | --- | --- | --- |
|  | Active | Healing | Scar |  |
| Depth of invasion |  |  |  | **<0.001** |
| Mucosa (T1a) | 118 (36.1) | 408(57.3) | 120(74.1) |  |
| Submucosa (T1b) | 209 (63.9) | 304 (42.7) | 42 (25.9) |  |
| Lymphovascular invasion | 50 (15.3) | 86 (12.1) | 15 (9.3) | 0.137 |
| Perineural invasion | 15 (4.6) | 29 (4.1) | 5 (3.1) | 0.732 |
| Lymph node metastasis | 58 (17.7) | 85 (11.9) | 9 (5.6) | **<0.001** |
