# Supplementary material for: The Implications of Endoscopic Ulcer in Early Gastric Cancer: Can We Predict Clinical Behaviors from Endoscopy?
Source: PLoS One. 2016 Oct 14;11(10):e0164339. doi: 10.1371/journal.pone.0164339 (PMC5065238; doi:10.1371/journal.pone.0164339)
Supplement: S5 Table — (DOCX) [file pone.0164339.s005.docx]

**S5 table.** Univariate and multivariate analysis of risk factors for lymph node metastasis in differentiated-type gastric cancer (n = 1,669)

|  | **Lymph node metastasis** | | | **Logistic regression model (including ulcer)** | | | **Logistic regression model (including ulcer stage)** | | |
| --- | --- | --- | --- | --- | --- | --- | --- | --- | --- |
| N (%) | **Presence, n = 178** | **Absence, n= 1,491** | ***P*** | **Odds ratio** | **95% CI** | ***P*** | **Odds ratio** | **95% CI** | ***P*** |
| Age >40 (year) | 169 (94.9) | 1,439 (96.5) | 0.292 |  |  |  |  |  |  |
| Male | 131 (73.6) | 1,142 (76.6) | 0.374 |  |  |  |  |  |  |
| Tumor location |  |  | 0.987 |  |  |  |  |  |  |
| Upper | 19 (10.7) | 162 (10.9) |  |  |  |  |  |  |  |
| Middle | 24 (13.5) | 195 (13.1) |  |  |  |  |  |  |  |
| Lower | 135 (75.8) | 1,134 (76.1) |  |  |  |  |  |  |  |
| **Ulcer** |  |  | **<0.001** |  |  |  | - |  |  |
| **Presence** | 143 (80.3) | 1000 (67.1) |  | 1.575 | 0.943-2.631 | 0.082 |  |  |  |
| Absence | 35 (19.7) | 491 (32.9) |  | 1 |  |  |  |  |  |
| **Ulcer stage** |  |  | **<0.001** | - |  |  |  |  |  |
| **Active stage** | 67 (47.2) | 299 (29.9) |  | - |  |  | 2.934 | 1.052-8.179 | **0.040** |
| Healing stage | 70 (49.3) | 599 (59.9) |  | - |  |  | 1.791 | 0.648-4.950 | 0.261 |
| Scar stage | 5 (3.5) | 102 (10.2) |  | - |  |  | - |  |  |

| **Gross type** |  |  | **< 0.001** |  |  |  |  |  |  |
| --- | --- | --- | --- | --- | --- | --- | --- | --- | --- |
| Elevated | 64 (36.0) | 367 (24.6) |  | 1.929 | 1.079-3.450 | 0.027 | 2.150 | 1.065-4.340 | **0.033** |
| Flat | 22 (12.4) | 414 (27.8) |  | 1 |  |  | - |  |  |
| Depressed | 92 (51.7) | 710 (47.6) |  | 1.547 | 0.900-2.658 | 0.114 | 1.493 | 0.816-2.73 | 0.194 |
| Lauren classification |  |  | 0.418 |  |  |  |  |  |  |
| Intestinal | 168 (94.4) | 1,437 (96.4) |  |  |  |  |  |  |  |
| Diffuse | 3 (1.7) | 15 (1.0) |  |  |  |  |  |  |  |
| Mixed | 7 (3.9) | 39 (2.6) |  |  |  |  |  |  |  |
| **Tumor diameter≥30 (mm)** | 80 (44.9) | 466 (31.3) | **< 0.001** | 1.271 | 0.883-1.829 | 0.198 | 1.271 | 0.845-1.912 | 0.249 |
| **Depth of invasion** |  |  | **< 0.001** |  |  |  |  |  |  |
| Mucosa | 8 (4.5) | 778 (52.2) |  | 1 |  |  | 1 |  |  |
| Submucosa | 170 (95.5) | 713 (47.8) |  | 10.984 | 5.254-22.962 | **<0.001** | 9.976 | 4.260-23.364 | **<0.001** |
| **Lymphovascular invasion** | 101 (56.7) | 124 (8.3) | **<0.001** | 7.201 | 4.989-10.393 | **< 0.001** | 5.727 | 3.786-8.664 | **< 0.001** |
| **Perineural invasion** | 5 (2.8) | 23 (1.5) | 0.214 |  |  |  |  |  |  |
