# Supplementary material for: The Implications of Endoscopic Ulcer in Early Gastric Cancer: Can We Predict Clinical Behaviors from Endoscopy?
Source: PLoS One. 2016 Oct 14;11(10):e0164339. doi: 10.1371/journal.pone.0164339 (PMC5065238; doi:10.1371/journal.pone.0164339)
Supplement: S6 Table — (DOCX) [file pone.0164339.s006.docx]

**S6 table.** Univariate and multivariate analysis of risk factors for lymph node metastasis in undifferentiated-type gastric cancer (n = 1,601)

|  | **Lymph node metastasis** | | | **Logistic regression model (including ulcer)** | | | **Logistic regression model (including ulcer stage)** | | |
| --- | --- | --- | --- | --- | --- | --- | --- | --- | --- |
| N (%) | **Presence, n= 166** | **Absence, n= 1,435** | ***P*** | **Odds ratio** | **95% CI** | ***P*** | **Odds ratio** | **95% CI** | ***P*** |
| Age >40 (year) | 135 (81.3) | 1,233 (85.9) | 0.130 |  |  |  |  |  |  |
| Male | 89 (53.6) | 755 (52.6) | 0.870 |  |  |  |  |  |  |
| Tumor location |  |  | 0.614 |  |  |  |  |  |  |
| Upper | 14 (8.4) | 150 (10.5) |  |  |  |  |  |  |  |
| Middle | 37 (22.3) | 346 (24.1) |  |  |  |  |  |  |  |
| Lower | 115 (69.3) | 939 (65.4) |  |  |  |  |  |  |  |
| **Ulcer** |  |  | **<0.001** |  |  |  |  |  |  |
| **Presence** | 151 (91.0) | 1,049 (73.1) |  | 2.067 | 1.093-3.912 | **0.026** |  |  |  |
| Absence | 15 (9.0) | 386 (26.9) |  | 1 |  |  |  |  |  |
| **Ulcer stage** |  |  | **< 0.001** |  |  |  |  |  |  |
| Active stage | 58 (38.2) | 269 (25.6) |  |  |  |  | 2.534 | 1.138-5.642 | **0.023** |
| Healing stage | 85 (55.9) | 627 (59.8) |  |  |  |  | 1.943 | 0.901-4.191 | 0.090 |
| Scar stage | 9 (5.9) | 153 (14.6) |  |  |  |  | 1 |  |  |

| **Gross type** |  |  | **0.007** |  |  |  |  |  |  |
| --- | --- | --- | --- | --- | --- | --- | --- | --- | --- |
| Elevated | 24 (14.5) | 160 (11.1) |  | 0.996 | 0.524-1.891 | 0.990 | 0.819 | 0.390-1.721 | 0.598 |
| Flat | 34 (20.5) | 456 (31.8) |  | 1 |  |  | 1 |  |  |
| Depressed | 108 (65.1) | 819 (57.1) |  | 1.160 | 0.715-1.880 | 0.548 | 0.921 | 0.560-1.516 | 0.746 |
| **Lauren classification** |  |  | **<0.001** |  |  |  |  |  |  |
| Intestinal | 35 (21.1) | 199 (13.9) |  | 1.306 | 0.816-2.090 | 0.265 | 1.246 | 0.757-2.049 | 0.387 |
| Diffuse | 107 (64.5) | 1,138 (79.3) |  | 1 |  |  | 1 |  |  |
| Mixed | 24 (14.5) | 98 (6.8) |  | 1.405 | 0.799-2.469 | 0.237 | 1.379 | 0.757-2.512 | 0.293 |
| **Tumor diameter ≥30 (mm)** | 85 (51.2) | 436 (30.4) | **< 0.001** | 1.869 | 1.300-2.688 | **0.001** | 1.963 | 1.339-2.877 | **0.001** |
| **Depth of invasion** |  |  | **< 0.001** |  |  |  |  |  |  |
| Mucosa | 32 (19.3) | 897 (62.5) |  | 1 |  |  | 1 |  |  |
| **Submucosa** | 134 (80.7) | 538 (37.5) |  | 3.407 | 2.184-5.313 | **<0.001** | 2.857 | 1.776-4.596 | **< 0.001** |
| **Lymphovascular invasion** | 76 (45.8) | 87 (6.1) | **< 0.001** | 6.278 | 4.153-9.490 | **< 0.001** | 6.267 | 4.048-9.701 | **< 0.001** |
| **Perineural invasion** | 13 (7.8) | 36 (2.5) | **0.001** | 1.365 | 0.659-2.829 | 0.403 | 1.366 | 0.651-2.867 | 0.410 |
